# Supplementary material for: Three-dimensional printing of silica glass with sub-micrometer resolution
Source: Nat Commun. 2023 Jun 7;14:3305. doi: 10.1038/s41467-023-38996-3 (PMC10244462; doi:10.1038/s41467-023-38996-3)
Supplement: Supplementary file 1 — Supplementary Information [file 41467_2023_38996_MOESM1_ESM.pdf]

Supplementary Information

Three-dimensional printing of silica glass with sub-micrometer resolution

Po-Han Huang[1,†], Miku Laakso[1,†], Pierre Edinger[1], Oliver Hartwig[2], Georg S. Duesberg[2], Lee-Lun Lai[1], Joachim Mayer[3], Johan Nyman[4], Carlos Errando-Herranz[1], Göran Stemme[1], Kristinn B. Gylfason[1], and Frank Niklaus[1,*]

[1] Division of Micro and Nanosystems, School of Electrical Engineering and Computer Science, KTH Royal Institute of Technology, Stockholm 10044, Sweden

[2] Institute of Physics, Faculty of Electrical Engineering and Information Technology (EIT 2), Universität der Bundeswehr Munich, Neubiberg 85579, Germany

[3] Central Facility for Electron Microscopy (GFE), RWTH Aachen University, Aachen 52074, Germany

[4] Department of Physics, Chemistry and Biology (IFM), Linköping University, Linköping 58183, Sweden

[*] Corresponding author: Frank Niklaus (frank@kth.se)

[†] These authors contributed equally to this work

The Supplementary Information includes:

Supplementary Tables 1-3

Supplementary Figures 1-15

References

Supplementary Table 1. Summary of energy-dispersive X-ray spectroscopy (EDS) results. The results measured from a lamella sliced from a 3D-printed silica glass sample. The values are given in atomic percentages and 11 points were measured in total.

Statistic	Silicon	Oxygen	Carbon
Average	29.80	70.00	0.21
Standard deviation	1.36	1.55	0.31
Maximum	44.94	71.31	0.68
Minimum	28.69	65.37	0.00

Supplementary Table 2. Identification of the observed Raman shifts.

Peak [reference]	Observed Raman shift (cm^{-1})
4-membered ring[1]	490
3-membered ring[1]	605
Si-OH[2]	975
C-H (asymmetric bending)[3]	1,455
C=C[3]	1,620
H_2O (bending mode)[2]	1,620
Si-H[4]	2,260
C-H (stretching)[5]	2,935
H_2O (O-H stretching)[2]	3,450
Unspecified SiOH species[2]	3,598
SiO-H stretching[2]	3,665

Supplementary Table 3. Summary of nanoindentation characterization results. Measurement results of our 3D-printed microplates, along with reference samples consisting of laser-ablated fused-silica microplates and flat fused-silica substrates. All sample types were measured without, and with annealing at 900 °C. For each sample, three measurements were performed. The measured hardness and reduced elastic modulus values are in the format of average ± standard deviation of the three measurements of each sample.

Sample	Hardness (GPa)	Reduced elastic modulus (GPa)
3D-printed microplate without annealing	2.4 ± 0.2	40 ± 2
3D-printed microplate annealed at 900 °C	7.7 ± 0.6	75 ± 2
Laser-ablated fused-silica microplate	7.4 ± 0.1	63.8 ± 0.6
Laser-ablated fused-silica microplate annealed at 900 °C	8.1 ± 0.5	66 ± 1
Flat fused-silica substrate	9.44 ± 0.05	69.7 ± 0.7
Flat fused-silica substrate annealed at 900 °C	9.62 ± 0.03	71.0 ± 0.1

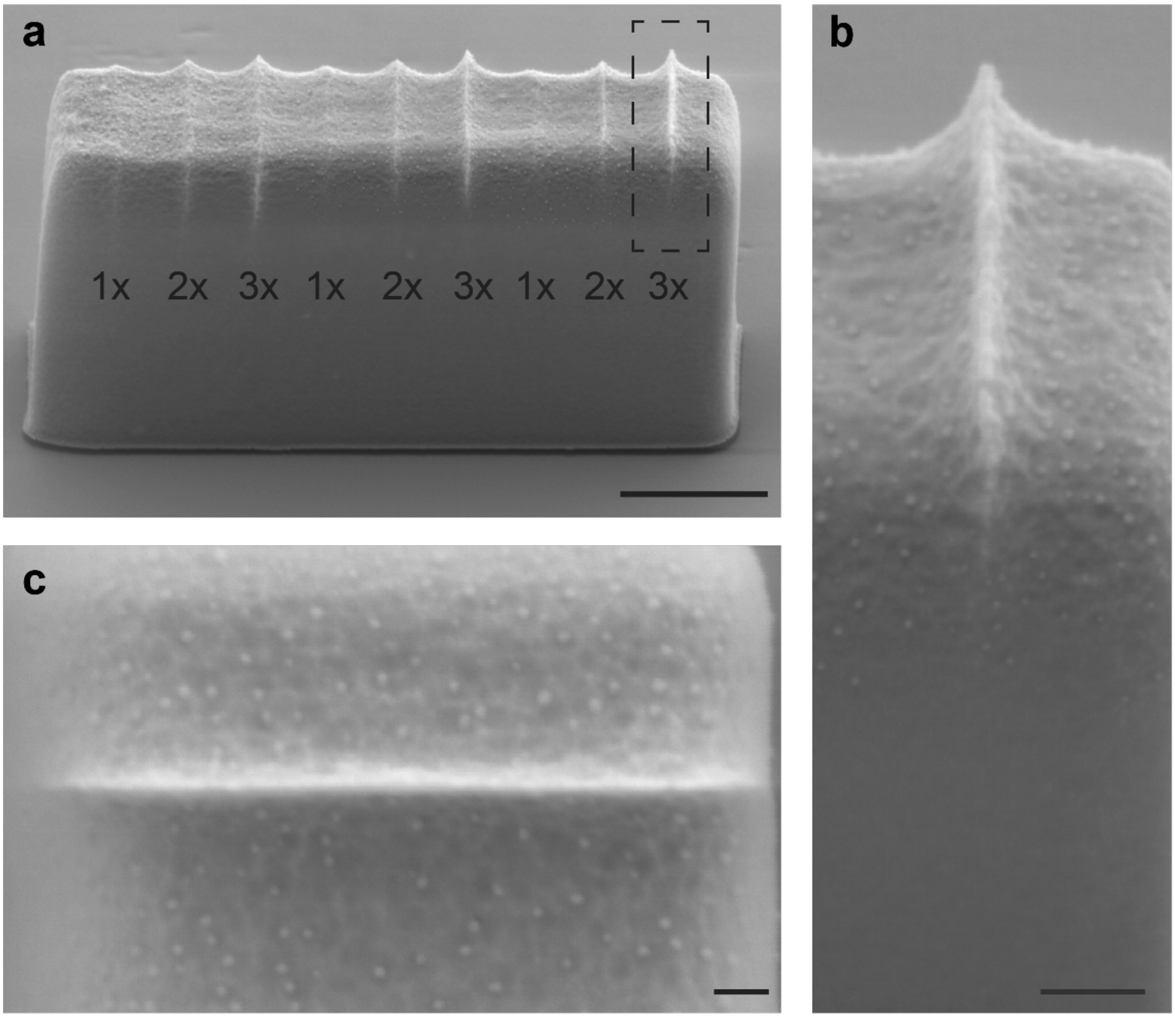

Supplementary Fig. 1. SEM images of printed single lines used for extracting the dimensions of a single voxel. **a**, Three sets with three printed single lines on a printed solid support block. Each line was scanned with laser by one, two, or three times indicated by the labels. The distance between the lines and the top surface of the block is fixed in one set and is increased from the leftmost set to the rightmost set. The line that best demonstrates the voxel dimensions is marked by a dashed rectangle. The image was taken from a 45-degree angle above the substrate plane (scale bar, 1 μm). **b**, Enlarged view of the line marked by the dashed rectangle in **a**, taken from the same angle (scale bar, 100 nm). **c**, Enlarged top view of the same line marked in **a** (scale bar, 200 nm).

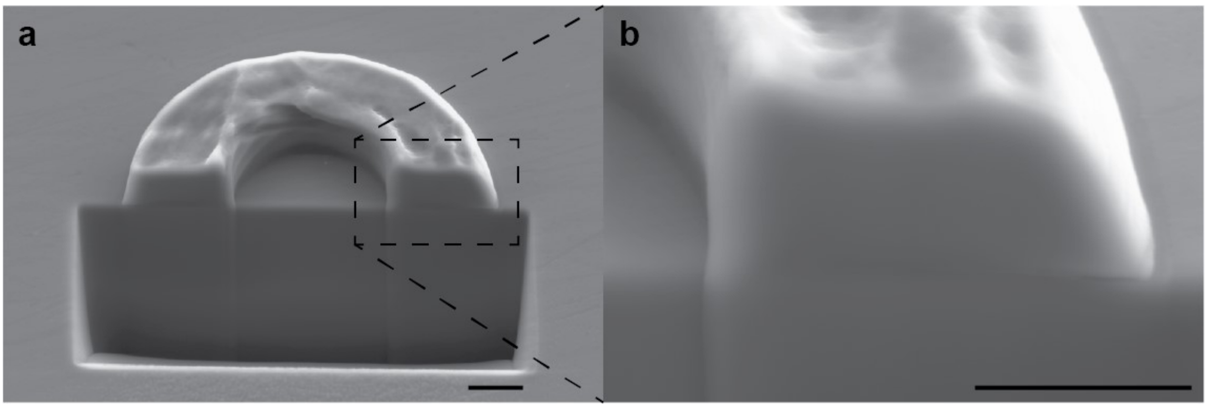

Supplementary Fig. 2. Cross-sectional SEM images reveal as-printed silica glass to be homogenous. a, An SEM image of a laser-written silica glass structure on a silica glass substrate. The printed structure was cross-sectioned by focused ion beam milling, extending down into the substrate. **b,** An enlarged view of a part of the cross-section. No porosity is visible in the cross-sections. (Scale bars, 1 μm)

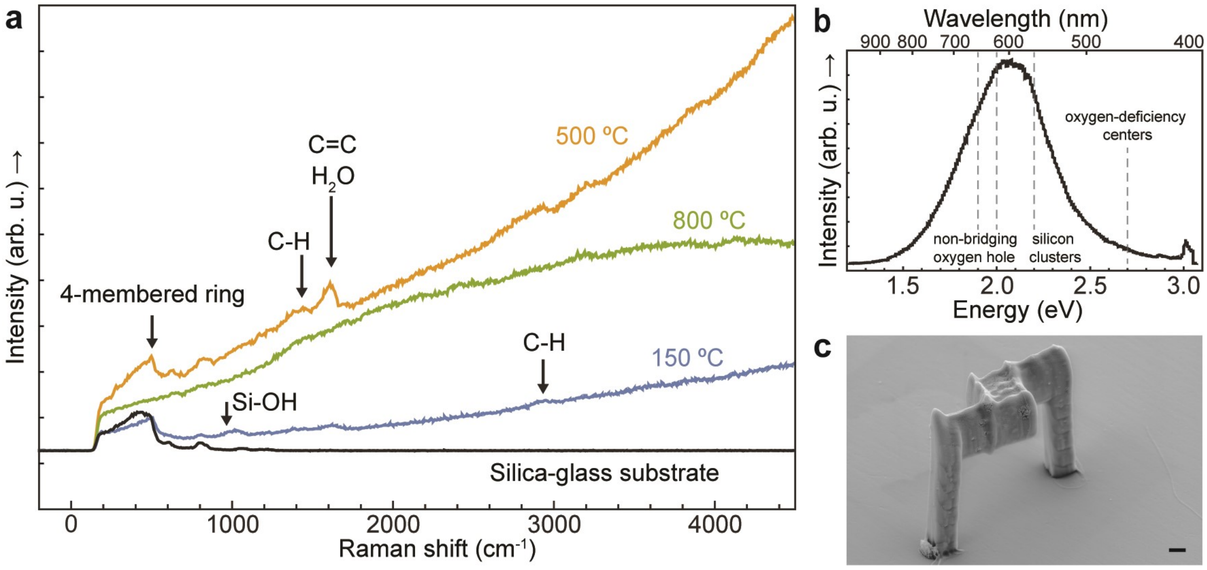

Supplementary Fig. 3. Raman structures and spectra together with the photoluminescence spectrum. a, Raman spectra of our 3D-printed glass structures after annealing at three different temperatures, and a Raman spectrum of a commercial silica glass substrate for comparison. A strong sloped photoluminescent background is present on top of the Raman peaks in the spectra. **b,** Photoluminescence spectrum of a 3D-printed glass structure annealed at 500 ºC, covering the entire spectral range of the sloped background observed in **a**. Possible origins of the photoluminescence background are labelled at their corresponding spectral positions. The peaks around 3 eV originate from Raman scattering. **c,** Example of 3D-printed glass structures used in our Raman measurements (scale bar, 1 µm).

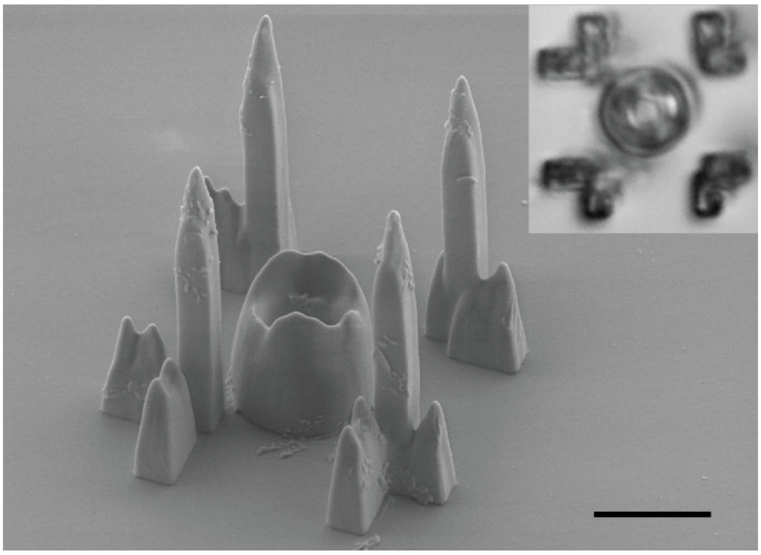

Supplementary Fig. 4. Reference images of the 3D-printed glass ring without a suspended glass plate above the ring structure. SEM image of the 3D-printed silica glass ring without a suspended plate, as a reference for the images of the ring with a suspended plate (Fig. 2c-f), and the inset image shows its optical microscope top-view. To include the potential effects of the supporting legs of the plate in Fig. 2c-f on the view of the ring, identical legs (but without plate) were printed around the reference ring (scale bar, 5 μm).

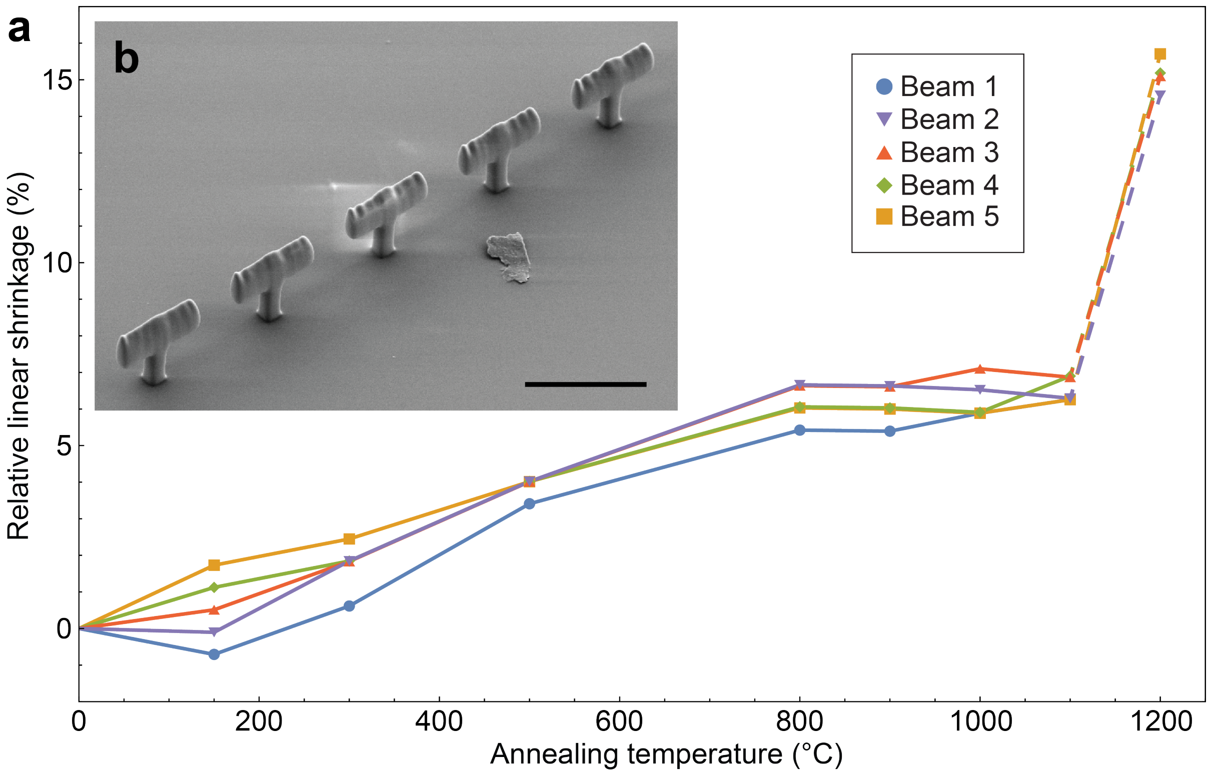

Supplementary Fig. 5. Individual relative linear shrinkages of the five 3D-printed T-shaped silica-glass test structures. a, The shrinkages as a function of annealing temperature obtained by measuring the length change of the horizontal beam of each structure. The extraordinary reduction in the length of the test structures after annealing at 1200 °C is a result of reflow and surface smoothening of the test structures and does not represent the material shrinkage. This is indicated by the dashed lines. **b,** SEM image of the measured structures after annealing at 1200 °C (scale bar, 10 µm).

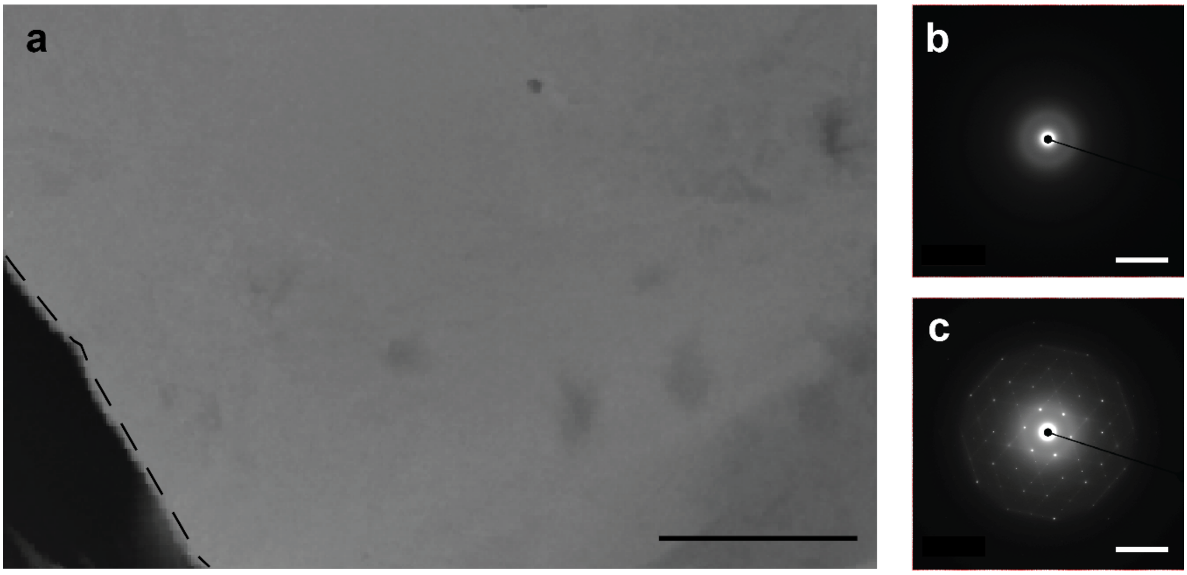

Supplementary Fig. 6. TEM characterization of 3D-printed glass after annealing at 900 °C. a, High-resolution TEM image of a lamella sliced from the annealed 3D-printed glass showing the substantial amorphous phase embedded with crystalline dark spots. The interface between the glass and surface metal is marked with a dashed line (scale bar, 1 μm). **b,** Electron diffraction pattern of the amorphous region in **a** which is clearly different from the diffraction pattern of the as-printed glass before annealing in Fig. 1k in the ring radius. This indicates that there was an amorphous-amorphous phase transition during annealing (scale bar: 5 nm^{-1}). **c,** Electron diffraction pattern of the dark spots in **a** showing a crystalline pattern on top of the amorphous pattern. The d-spacing values identified in the crystalline pattern are 4.276 Å, 2.453 Å, 2.127 Å, 1.611 Å, and 1.18 Å which correspond well to the values for α-quartz. This shows that the 3D-printed glass remained substantially amorphous after annealing at 900 °C, while local nucleation and growth of crystalline α-quartz regions started during the annealing (scale bar, 5 nm^{-1}).

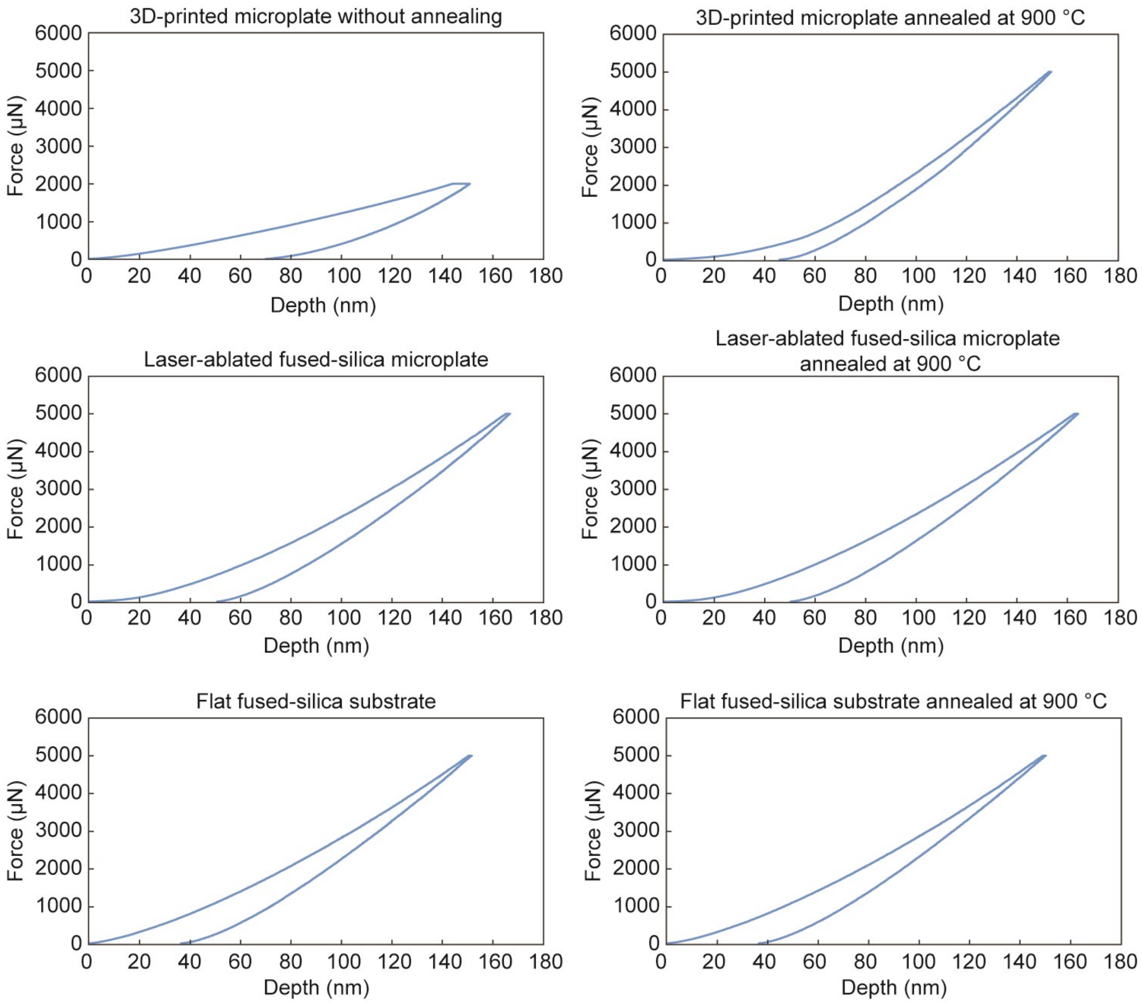

Supplementary Fig. 7. Nanoindentation characterization of the 3D-printed glass and the fused silica glass reference samples. Loading-unloading curves of the samples listed in Supplementary Table 3. One of the measured three curve from each sample is plotted to demonstrate the behavior of the samples in the indentation experiments.

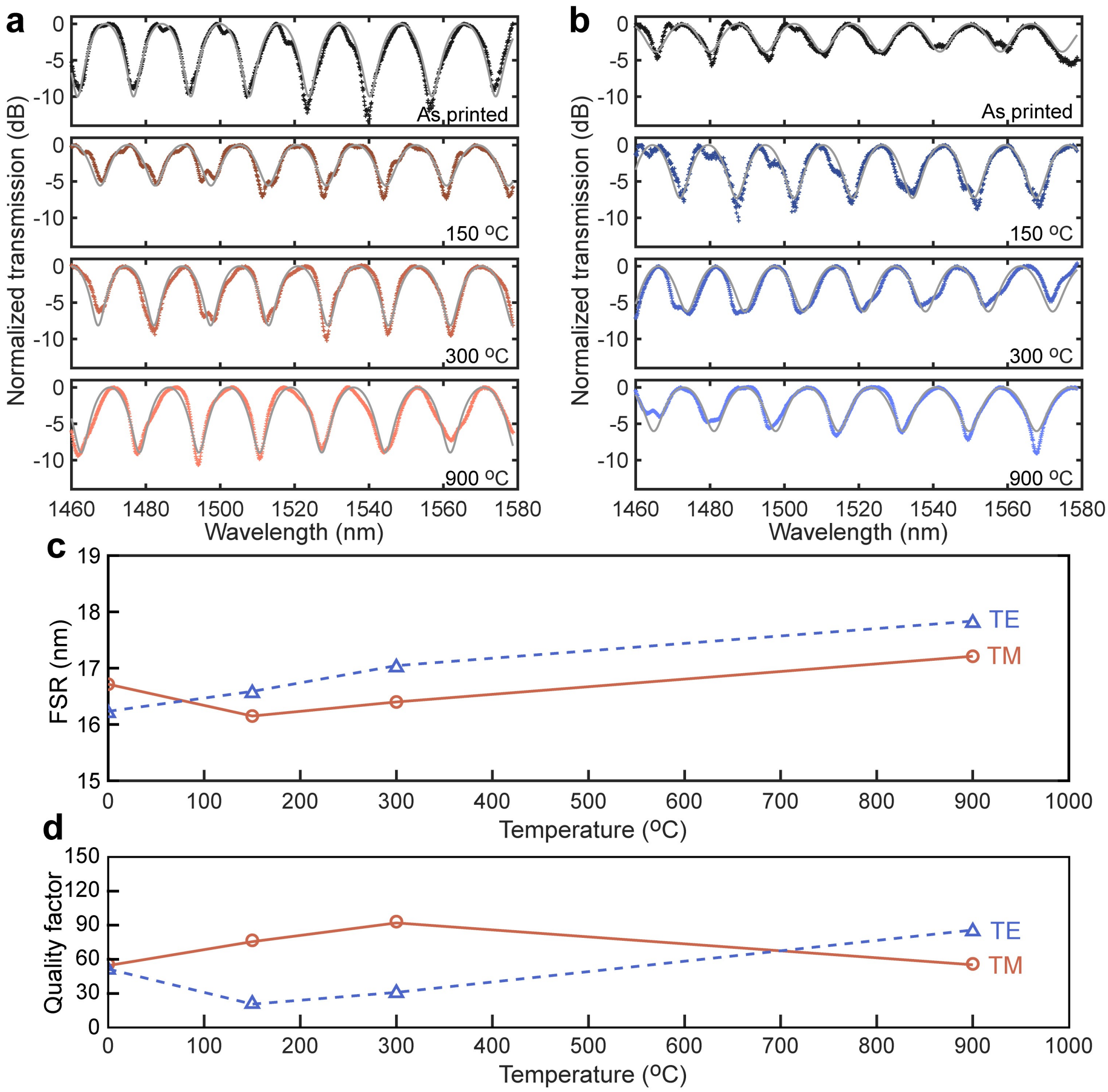

Supplementary Fig. 8. Transmission, free spectral range (FSR), and quality factor of the microtoroid resonator. a, b, Measured transmission spectra of the microtoroid resonator using transverse magnetic (TM) and transverse electric (TE) input light, respectively. The transmission spectra were measured for the as-printed resonator and after annealing at different temperatures. The measured spectra were fitted with an all-pass, single-mode ring resonator model, shown as grey lines. **c, d,** The FSR and the quality factor of the resonator after annealing at different temperatures, respectively. The presented values were extracted from the fitted transmission spectra shown in **a** and **b**. The FSR is shown for a wavelength of 1550 nm while the quality factor corresponds to an average value over the wavelength range.

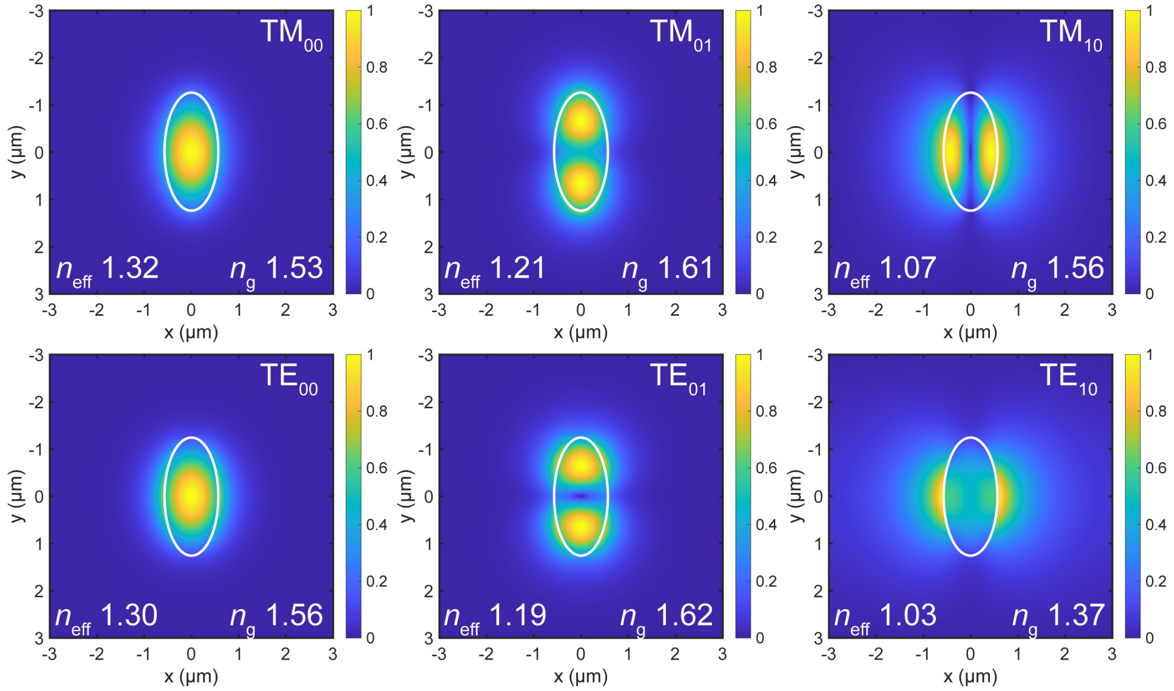

Supplementary Fig. 9. Simulated, normalized magnitudes of the electrical field of the eigenmodes supported by the 3D-printed waveguides in the microtoroid resonator. SEM images of the resonator system are shown in Fig. 3a, b. The simulated effective mode indexes n_{eff} and group indexes n_{g} are marked for each eigenmode, at a wavelength of 1550 nm. The simulations were made using Lumerical MODE 2020a software, for straight waveguides.

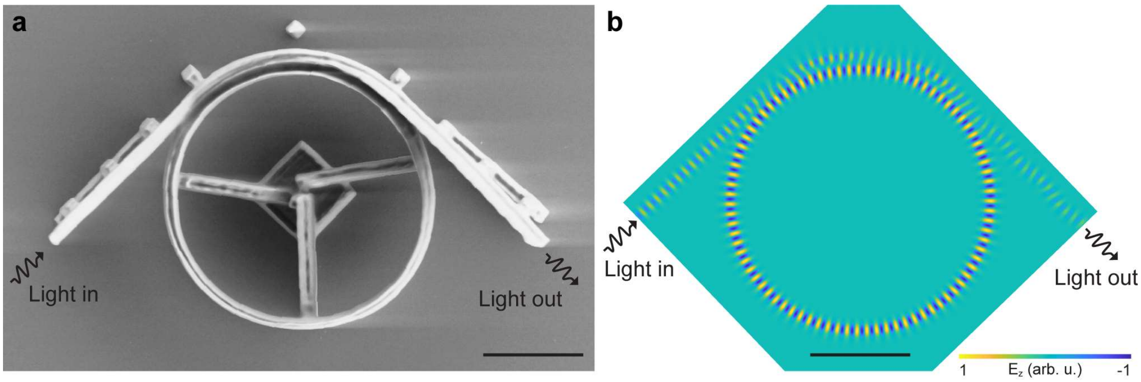

Supplementary Fig. 10. Simulated vertical component of the electrical field at resonance in the microtoroid resonator. a, Original SEM top-view of the microtoroid resonator shown in Fig. 3b **b**, Original graph of the simulated vertical component of the electrical field (E_z) at resonance when injecting TM_{00} into the resonator shown in Fig. 3b, displayed at the same view and size as **a**. (Scale bars, 10 µm)

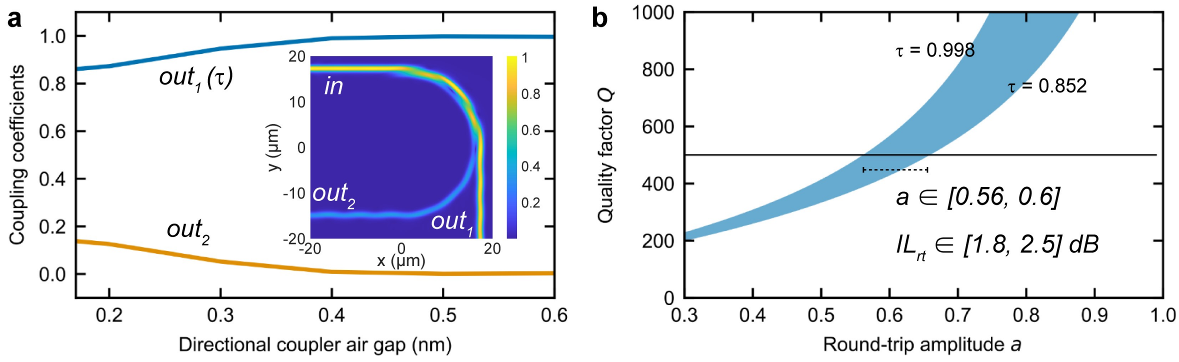

Supplementary Fig. 11. Estimate of the round-trip loss based on measurements and simulations. a, Lumerical 3D FDTD simulation of the directional coupler portion of the ring resonator to obtain the coupling coefficients as a function of the air gap between the ring and the bus waveguide. In the 3D FDTD simulation a rectangular waveguide with a width of 1.2 µm and a height of 2.5 µm was assumed. **b,** Extracted round-trip amplitude a (and corresponding round-trip loss IL_{rt} in dB) using Equation (4), the simulated self-coupling coefficient τ, and the maximum measured Q of 500.

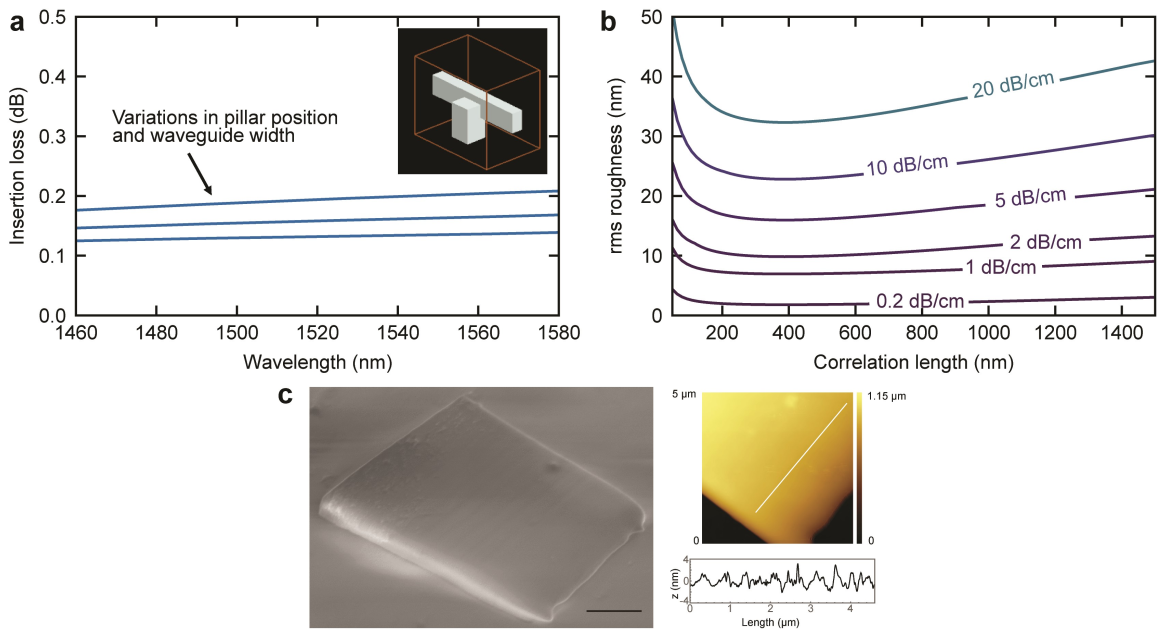

Supplementary Fig. 12. Analysis of loss contributions to the round-trip loss of the microtoroid resonator. a, Simulations using Lumerical 3D FDTD of a supporting pillar, for different positions with respect to the waveguide, and different waveguide widths. **b,** Losses due to sidewall scattering can be estimated analytically using the sidewall roughness amplitude and correlation length[6]. With a measured sidewall roughness of below 1 nm shown in **c**, the 3D-printed waveguides feature low propagation losses due to the sidewall roughness. **c,** Surface roughness characterization of the sidewall of a representative 3D-printed structure by atomic force microscopy from the top after the structure was intentionally overturned, showing a root-mean-square sidewall surface roughness of about 1 nm (scale bar, 2 µm).

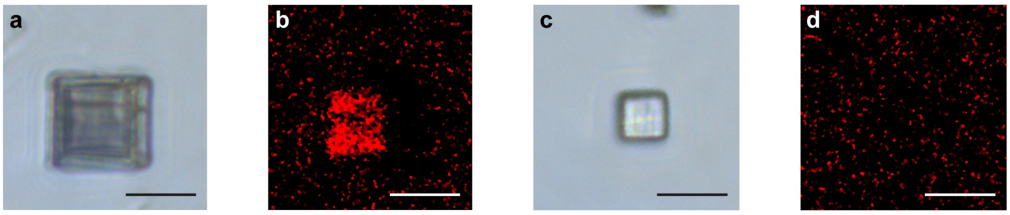

Supplementary Fig. 13. Selective generation of silicon nanocrystals in 3D-printed cubes after annealing at 1200 °C. a, b, Original optical microscope top-view image and the photoluminescence image at the same view taken from the shell-exposed cube shown in Fig. 3d, respectively. **c, d,** Original optical microscope top-view image and the photoluminescence image at the same view taken from the fully exposed cube shown in Fig. 3e, respectively. (Scale bars, 10 μm)

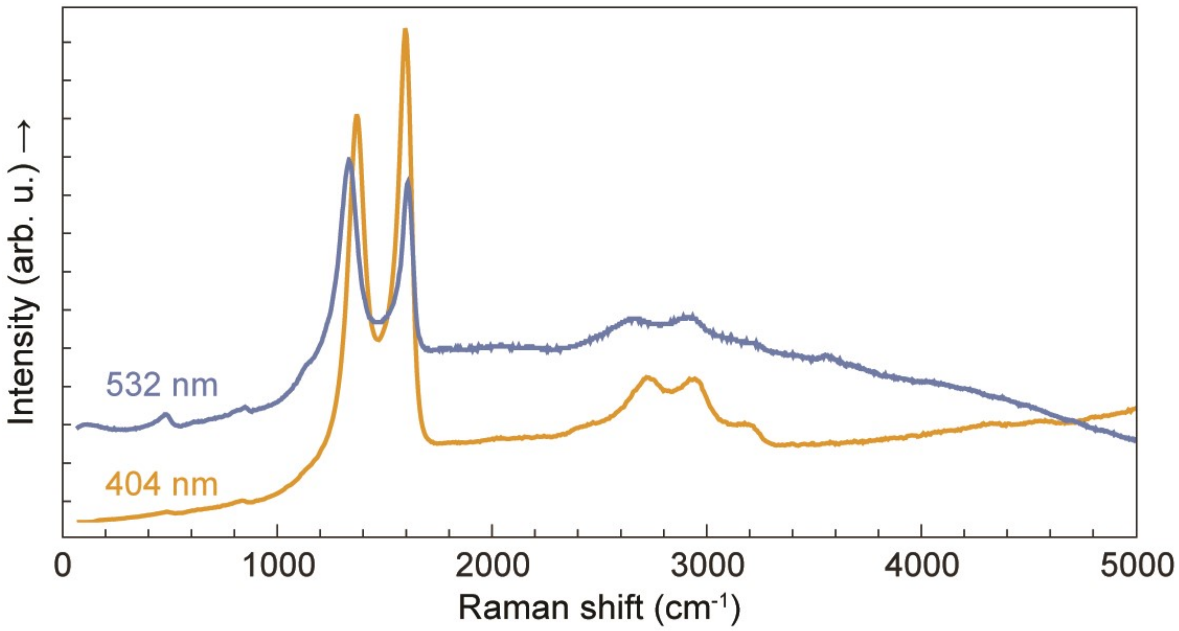

Supplementary Fig. 14. Confirmation of the Raman-scattering origin of the side peaks in the photoluminescence spectrum of the shell-exposed cube. Spectra shown here were collected from the core of the shell-exposed cube in Fig. 3d under excitation of two different lasers with wavelengths of 405 nm and 532 nm, respectively. The peaks in both spectra appeared at the identical shift positions, indicating their cause as Raman-scattering and not photoluminescence.

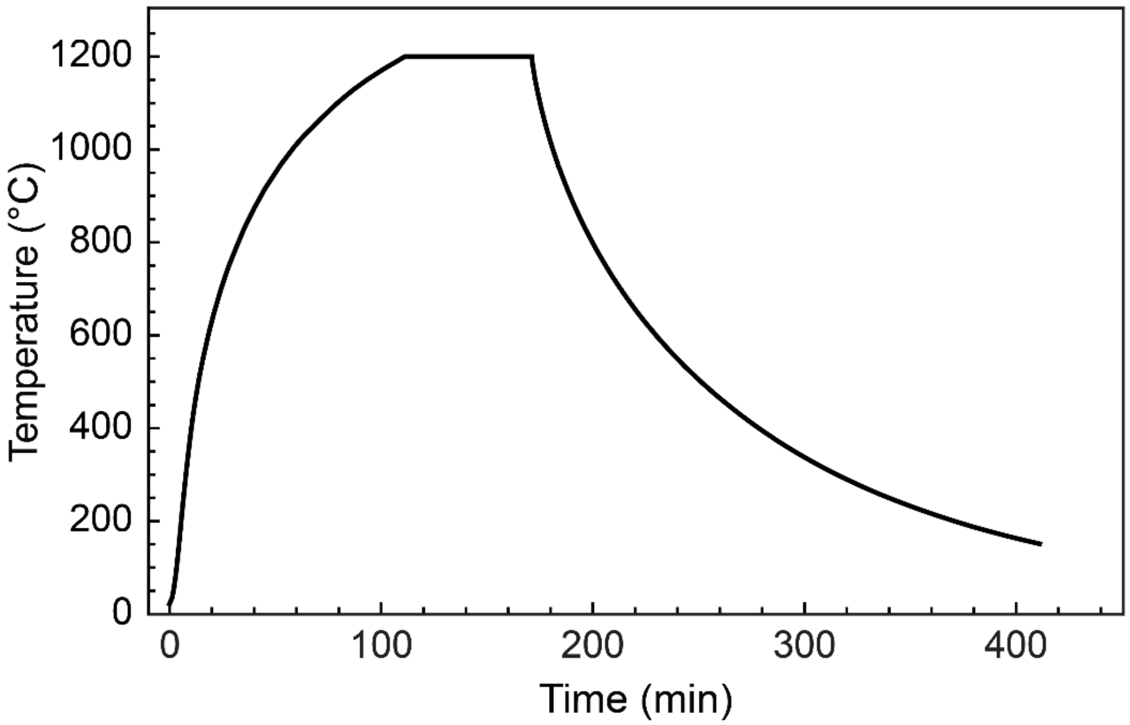

Supplementary Fig. 15. Temperature ramping profile of the oven the oven in the 1200 °C annealing process. The temperature ramping profile during heating was dictated by the maximum heating power of the oven. The profile during cooling was a result of natural cooling defined by the thermal dissipation from the oven to the room-temperature surrounding environment. This temperature ramping procedure was used in all annealing experiments.

References

1. Chan, J. W. W., Huser, T. R. R., Risbud, S. H. H. & Krol, D. M. M. Modification of the fused silica glass network associated with waveguide fabrication using femtosecond laser pulses. *Appl. Phys. A Mater. Sci. Process.* **76**, 367–372 (2003).
2. McMillan, P. F. & Remmele, R. L. J. Hydroxyl sites in SiO2 glass: A note on infrared and Raman spectra. *Am. Mineral.* **71**, 772–778 (1986).
3. Sulca, N. M., Lungu, A., Garea, S. A. & Iovu, H. Monitoring the synthesis of new polymer nanocomposites based on different polyhedral oligomeric silsesquioxanes using Raman spectroscopy. *J. Raman Spectrosc.* **40**, 1634–1640 (2009).
4. Olynick, D. L., Cord, B., Schipotinin, A., Ogletree, D. F. & Schuck, P. J. Electron-beam exposure mechanisms in hydrogen silsesquioxane investigated by vibrational spectroscopy and in situ electron-beam-induced desorption. *Cit. J. Vac. Sci. Technol. B* **28**, 581 (2010).
5. Bertoluzza, A., Fagnano, C., Antonietta Morelli, M., Gottardi, V. & Guglielmi, M. Raman and infrared spectra on silica gel evolving toward glass. *J. Non. Cryst. Solids* **48**, 117–128 (1982).
6. Payne, F. P. & Lacey, J. P. R. A theoretical analysis of scattering loss from planar optical waveguides. *Opt. Quantum Electron.* **26**, 977–986 (1994).
